# A Note on Graphs with Prescribed Orbit Structure

**DOI:** 10.3390/e21111118

**Published:** 2019-11-15

**Authors:** Abbe Mowshowitz, Matthias Dehmer, Frank Emmert-Streib

**Affiliations:** 1 Department of Computer Science, The City College of New York (CUNY), 138th Street at Convent Avenue, New York, NY 10031, USA; 2 Steyr School of Management, University of Applied Sciences Upper Austria, 4600 Wels, Austria; matthias.dehmer@umit.at; 3 Predictive Medicine and Data Analytics Lab, Department of Signal Processing, Tampere University of Technology, 33720 Tampere, Finland; frank.emmert.streib@gmail.com; 4 Institute of Biosciences and Medical Technology, Tampere University, 33520 Tampere, Finland

**Keywords:** graphs, automorphism groups, orbits, trees

## Abstract

This paper presents a proof of the existence of connected, undirected graphs with prescribed orbit structure, giving an explicit construction procedure for these graphs. Trees with prescribed orbit structure are also investigated.

## 1. Introduction

Quantitative measures of graph complexity, defined in terms of Shannon entropy, are typically based on a partition of the vertices or edges of a graph. One such partition of the vertices of a graph is related to symmetry structure. In this paper, we reverse the order of things and start by asking “is there a graph with a given entropy value”, which translates into “is there a graph whose symmetry structure produces a partition giving that entropy value”.

It has long been known that any partition of a positive integer can be realized as the orbit structure of the automorphism group of a weakly connected directed graph [1]. This structure provides the basis for certain quantitative measures of graph complexity. In particular, the set of orbits constitutes a partition of the vertices of a graph, thus defining an equivalence relation. An information theoretic measure based on this equivalence relation has been elaborated and refined as an index of graph complexity, see [2,3] for an overview. This is an example of a topological graph measure based on the automorphism group of a graph, and many such measures have been developed [4,5,6]. Investigations of the structural complexity of graphs and networks [7] have figured prominently in such development. Vertices in the same orbit are similar under graph automorphism and thus share many graph-theoretical properties [8]. Most of these measures depend on determining vertex or edge orbits [4,6]. Since automorphisms are permutations of the vertices that preserve adjacencies, a brute force approach, examining all n! permutations is not feasible. See [9,10] for discussion of orbit computation methods and heuristics.

This note focuses on the question of whether or not a given partition of a positive integer can be realized as the orbit sizes of the automorphism group of some graph. In particular, we present a proof of the existence of connected, undirected graphs whose automorphism groups have prescribed orbit structure. The proof proceeds by explicitly constructing the graphs in question. The component graphs in this construction are shown to have a minimal number of edges, and a special class of trees with prescribed orbit structures is also produced. The methods developed here can be used to construct graphs with an explicit degree of symmetry. In addition, the graphs so constructed can be used to calculate and interpret existing symmetry measures, see [4,5,6].

Most of the graph theoretic definitions needed for subsequent sections are given here; specialized definitions are introduced as needed. The terminology and notation is standard, see for example [11] for further details. An *undirected graph* G=(V,E) consists of a set V=V(G) of *vertices* and a set E=E(G) of unordered pairs of vertices called *edges*. An edge e∈E has the form e=u,v for vertices u,v∈V, u≠v; *u* and *v* are said to be *adjacent* in *G*. Thus, the graphs considered here have no loops, (i.e, edges of the form u,u), and no parallel edges.

The *degree* of a vertex is the number of vertices to which it is adjacent. An edge e=u,v is said to be *incident* to *u* and *v*. A *path* $P_{n}$ with *n* vertices is a sequence of n−1 edges $e_{1},\ldots ,e_{n-1}$ in which $e_{i}=u,v$ and $e_{i+1}=v,w,1\leq i\leq n-2$, and $e_{1}$ and $e_{n}$ have no vertex in common. The length of $P_{n}$ is n−1, the number of edges contained in it. If $e_{1}$ and $e_{n}$ are both incident to a common vertex, the sequence defines a *cycle* $C_{n-1}$ with n−1 vertices and n−1 edges. A *subgraph* S=(U,F) of a graph G=(V,E) is defined by U⊆V and F⊆E such that every edge in *F* is incident to vertices in *U*. A graph *G* is said to be *connected* if there is path between every pair of distinct vertices in *G*. If a graph *G* is not connected, it is said to be disconnected and consists of two or more connected subgraphs called *components*. A *tree* is a connected undirected graph with no cycles. The complete graph on *n* vertices, denoted by $K_{n}$, contains all the n2 pairs of distinct vertices in its edge set. The *complement* of a graph *G*, denoted by $\bar{G}$, has vertex set *V* and edge set $\bar{E}$ consisting of all the unordered pairs u,v (u,v∈V and u≠v), not in *E*, i.e., $\bar{E}=E\left(K_{n}\right)-E\left(G\right)$. Two graphs G=(U,E) and H=(V,F) are *isomorphic* if there is a bijection α mapping *U* to *V* such that for any e∈E with e=u,v, α(u),α(v)∈F. An *automorphism* of a graph *G* is an isomorphism of *G* to itself. The collection of automorphisms forms a group Aut(G) under composition. Vertices *u* and *v* are said to be *similar* if there exists an automorphism mapping *u* to *v*. An *orbit* of Aut(G) is the set of all vertices similar to a given vertex. The collection of orbits constitutes a partition of *V*. A graph *G* whose automorphism group consists of the identity alone is called an *identity graph*.

## 2. Existence Theorem

**Theorem** **1.** *Let n be a positive integer such that $n=\sum_{i=1}^{i=t}i\cdot k_{i}$, for some positive integer t, where $k_{i}$ is the number of values equal to i in the sum. If $k_{1}\leq 1$ or $k_{1}\geq 6$, then there exists an undirected, connected graph G whose automorphism group Aut(G) has t orbits of sizes $k_{1},\ldots ,k_{t}$, respectively.*

**Proof.**  The proof is by construction. First, we show how to form a disconnected, undirected graph *H* with the requisite orbit structure. The constructed graph *H* consists of disjoint, non-isomorphic subgraphs, each of which contributes a unique orbit to the automorphism group Aut(H). The complement $G=\bar{H}$ is then the desired result, since it is connected and has the same orbit structure as *H*. This follows from the well known facts that (1) the complement of a disconnected graph is connected, and (2) $Aut(\bar{G})$ is the same as Aut(G).

**Figure 1:**
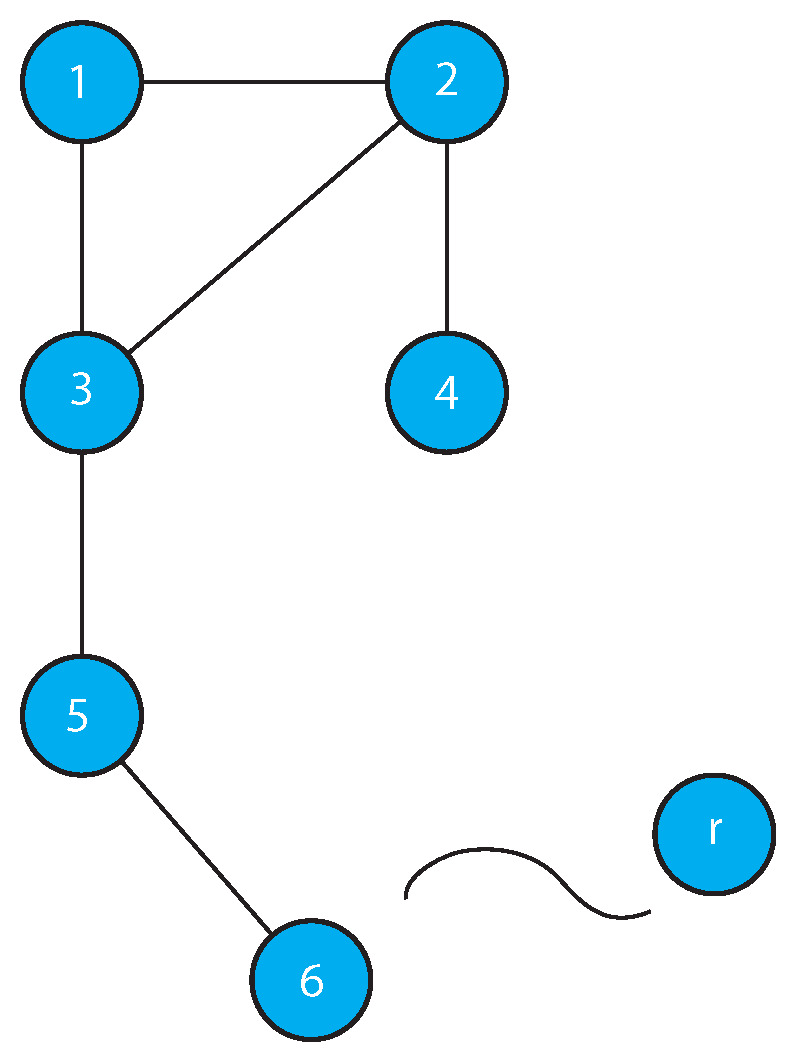
Identity Graphs.

If $k_{1}=0$, there is no need for an orbit of size 1 in *H*; if $k_{1}=1$, a single isolated vertex in *H* supplies the necessary orbit of size 1. Now, if $k_{1}\geq 6$, the component constructed as shown in Figure 1 (by attaching $k_{1}-6$ vertices successively starting at vertex 6 and continuing with each added vertex) has $k_{1}$ orbits of size 1. Note that the graph in Figure 1 consisting of six vertices is the smallest connected graph with an identity group. This is well known and can easily be confirmed by examining the relatively small set of undirected graphs with between 2 and 5 vertices. The construction (illustrated in the figure) produces successively larger connected graphs with identity group. Note that for $k_{1}\geq 7$, an alternative identity graph, namely, an identity tree can be constructed by adding a vertex joined to the third vertex from either end of a path of length $k_{1}-1$.

For $k_{2}\geq 1$, a path $P_{2k_{2}}$ of length $2k_{2}$ has automorphism group $Aut(P_{2k_{2}})$ with exactly $k_{2}$ orbits.

**Figure 2:**
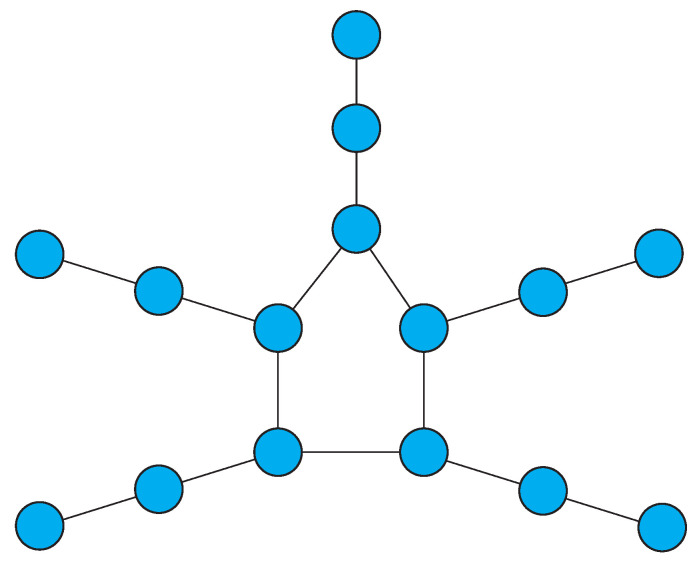
Augmented 5-cycle with three orbits.

The remaining components of *H*, providing orbits of sizes 3,···,t, are augmented cycles. A cycle $C_{i}$ with *i* vertices contributes exactly one orbit of size *i* provided there is no other copy of $C_{i}$ in the graph. To obtain $k_{i}$ orbits, $C_{i}$ is *augmented* by attaching a path of length $k_{i}-1$ to each of its vertices, resulting in a graph of the form $C_{i}^{*}$ as shown in Figure 2. The vertices in $C_{i}^{*}$ at the same distance from the point of attachment of each path belong to the same orbit, so that exactly $k_{i}$ orbits are contributed by such an augmented cycle.

The graph $G=\bar{H}$ satisfies the conclusion of the Theorem, for the reasons stated above in the explanation of the construction process. □

The case of $2\leq k_{1}\leq 5$ size 1 orbits can be realized if the $\sum_{i=2}^{i=t}k_{i}\geq 4$. A graph supplying the requisite orbits consists of a path joined to a vertex of a complete graph $K_{r},r\geq 3$. Two to five orbits of size 1 can be supplied by attaching a path of length 1–4. This construction also adds an orbit of size r−1 which necessitates having several orbits of sizes larger than 2.

Thus, it is clear that with the exception of some cases of size 1 orbits, any partition of a positive integer *n* can be realized as the orbit structure of the automorphism group of a connected, undirected graph.

## 3. Edge Minimal Graph Components

In this section we prove a related, but independent theorem, demonstrating that the components used in the construction of the disconnected graph *H* in the previous section have a minimal number of edges.

**Theorem** **2.** *Let n, $k_{i}$ and t be as given in the hypotheses of Theorem 1. The paths $P_{2k_{2}}$, augmented cycles, and identity graphs defined as components of H in the proof of Theorem 1, all have a minimal number of edges.*

**Proof.**  Each of the components of the graph *H* is connected, and obviously the paths $P_{2k_{2}}$ have the fewest edges possible for a connected graph. An augmented cycle $C_{i}^{*}$ can be reduced to a tree by the removal of a single edge, hence we need only show that (apart from $K_{2}$) no tree can have multiple orbits of the same size. Consider an augmented cycle $C_{t}^{*}$ with $k_{t}\geq 1$. When $k_{t}=1$, $C_{t}^{*}$ is a cycle of size *t* and thus its automorphism group has just one orbit of size *t*. Now, suppose $k_{t}\geq 1$, which implies that the automorphism group of the augmented cycle $C_{t}^{*}$ has more than one orbit of size *t*. Since all trees (apart from $K_{2})$ have at least two vertices of degree one and others of degree greater than one ([11]), it is not possible for the automorphism group of a tree to have only one orbit, since similar vertices must have the same degree. Claim: there is no tree with orbit structure the same as that of the augmented cycle. Suppose *T* is a tree with n=mr vertices, where m≥2 is the number of orbits of its automorphism group and r≥3 is the size of each orbit. Let $d_{i},1\leq i\leq m$ be the degree of the vertices in the *i*-th orbit. Since every tree has at least two vertices of degree 1, one of the $d_{i}$, say $d_{1}$, must be equal to 1. The sum of the degrees of the vertices in a tree is twice the number of edges, which for *T* is 2(mr−1). Suppose, $\sum_{i=1}^{i=m}rd_{i}=2\left(mr-1\right)$. Then $\sum_{i=2}^{i=m}d_{i}=2m-\frac{2}{r}$. Now, m≥2 and r≥3, so $2m-1<2m-\frac{2}{r}<2m$, contradicting the fact that $\sum_{i=2}^{i=m}d_{i}$ is a positive integer, thus proving the claim that the augmented cycle is edge minimal. Now observe that the identity graph with 6 vertices described above is the smallest identity graph, and that there exist identity trees with seven or more vertices, which completes the proof. □

## 4. Trees with Prescribed Orbit Structure

Under certain conditions, it is possible to construct a tree with prescribed orbit structure, as we prove in the following theorem.

**Theorem** **3.** *Let n, $k_{i}$ and t be as given in the hypotheses of Theorem 1, with $k_{i}\geq 2$ (2≤i≤t) and $k_{i}\leq k_{j}$ for i≤j. There exists a tree with n vertices whose automorphism group has $k_{i}$ orbits of size i, 2≤i≤t, and k1=∑i=2i=tki+ki2.*

**Figure 3:**
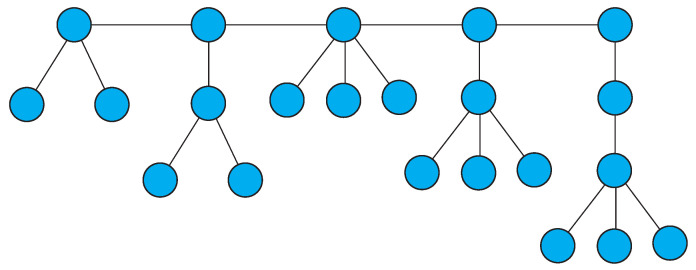
Tree with 14 orbits: nine of size 1, two of size 2, and three of size 3.

**Proof.**  A tree with the kind of orbit structure described in the Theorem is illustrated in Figure 3. The tree consists of a path of length t−1 (the number of orbits of size ≥2). For simplicity, suppose the $k_{i},2\leq i\leq t$ are listed in increasing order. For each $k_{i}$, attach a subsidiary path of length $j-1,1\leq j\leq k_{i}$ with *i* vertices of degree one, starting at one end of the main path. The total number of vertices in the tree is given by $\sum_{i=2}^{i=t}k_{i}\left(i+1\right)+\sum_{i=2}^{i=t}\sum_{j=1}^{j=k_{i}-1}j$. The subsidiary paths with requisite number of degree one vertices can be in any order. Note that each subsidiary path contributes precisely one orbit of size equal to the number of vertices of degree one at its termination, since no two of these paths are similar. The number of singleton orbits is $\sum_{i=2}^{i=t}k_{i}+\sum_{i=2}^{i=t}\sum_{j=1}^{j=k_{i}-1}j$. The second term in the summation equals ki2, which concludes the proof. □

## 5. Summary and Conclusions

In this note we have proved that a connected, undirected graph exists with orbit structure corresponding to virtually any partition of a positive integer. Moreover, the components used in the construction of such graphs are edge minimal. The special case of a tree whose automorphism group has orbits of mixed sizes was also constructed. This investigation of undirected graphs with prescribed orbit structure arose in connection with research dealing with a polynomial associated with the orbit structure of a graph [12]. A typical term in this so-called orbit polynomial [12] is of the form $cx^{n}$ where *n* is the size of an orbit and the coefficient *a* denotes the number of orbits of size *n*. The roots of this polynomial provide information about the structure of the graph, and it is of interest to know what graphs exist with a given set of orbit sizes.
